# The methylation signature of hepatocellular carcinoma trajectory based on pseudotime and chronological time for predicting precancerous patients

**DOI:** 10.1093/oncolo/oyae292

**Published:** 2024-11-26

**Authors:** Kang Li, Chaoran Zang, Yanan Zhao, Dandan Guo, Wanting Shi, Tingting Mei, Ang Li, Yonghong Zhang

**Affiliations:** Biomedical Information Center, Beijing You’An Hospital, Capital Medical University; Beijing 100069, People’s Republic of China; Beijing Key Laboratory (BZ0373), Beijing You’An Hospital, Capital Medical University, Beijing 100069, People’s Republic of China; Beijing Research Center for Respiratory Infectious Diseases, Beijing 100013, People’s Republic of China; Interventional Therapy Center for Oncology, Beijing You’An Hospital, Capital Medical University, Beijing 100069, People’s Republic of China; Hepatobiliary Pancreatic Center Department, Beijing Tsinghua Changgung Hospital Affiliated to Tsinghua University, Beijing 102218, People’s Republic of China; Interventional Therapy Center for Oncology, Beijing You’An Hospital, Capital Medical University, Beijing 100069, People’s Republic of China; Department of Minimal-Invasive Intervention, The Affiliated Cancer Hospital of Zhengzhou University & Henan Cancer Hospital, Zhengzhou 450008, People’s Republic of China; Interventional Therapy Center for Oncology, Beijing You’An Hospital, Capital Medical University, Beijing 100069, People’s Republic of China; Interventional Therapy Center for Oncology, Beijing You’An Hospital, Capital Medical University, Beijing 100069, People’s Republic of China; Interventional Therapy Center for Oncology, Beijing You’An Hospital, Capital Medical University, Beijing 100069, People’s Republic of China; Biomedical Information Center, Beijing You’An Hospital, Capital Medical University; Beijing 100069, People’s Republic of China; Beijing Key Laboratory (BZ0373), Beijing You’An Hospital, Capital Medical University, Beijing 100069, People’s Republic of China; Beijing Research Center for Respiratory Infectious Diseases, Beijing 100013, People’s Republic of China; Interventional Therapy Center for Oncology, Beijing You’An Hospital, Capital Medical University, Beijing 100069, People’s Republic of China

**Keywords:** hepatocellular carcinoma trajectory, precancerous patients, methylation, predictive nomogram

## Abstract

**Background:**

Early screening of hepatocellular carcinoma (HCC) is strongly recommended for hepatitis B virus (HBV)-infected patients. We aimed to develop and validate a predictive nomogram based on HCC occurrence trajectory for screening precancerous patients with HCC.

**Methods:**

Peripheral blood mononuclear cells (PBMC) samples from 22 patients with HCC with their precancerous stage (*n* = 55) and 18 healthy controls were measured using HumanMethylation EPIC BeadChip assay. HCC trajectory was assessed by pseudotime based on TimeAx algorithm and chronological time. The 43 candidate CpG sites were selected from the methylation signature and measured using multiplex bisulfite sequencing in a retrospective cohort of HBV-infected patients (*n* = 604). A 5-CpG-classifier was built using the LASSO Cox regression model, based on the association between the methylation level of every CpG and the duration from enrollment to HCC occurrence of individual patient. We validated the risk stratification and predictive accuracy of this classifier in both the primary cohort (*n* = 300) and independent validation cohort (*n* = 304).

**Results:**

Pseudotime and chronological time of HCC trajectory analysis revealed that the PD-1/PD-L1 pathway underwent changes in the precancerous stage. Based on the trajectory of methylation signature, we built a 5-CpG-classifier which remained powerful and independent predictive efficiency after stratified analysis by clinicopathological risk factors in both primary cohort and independent validation cohort. A predicting nomogram including the 5-CpG-classifier was constructed after multivariate analysis. One-year cumulative hazard of HCC in low- and high-risk groups of HBV-infected patients was 3.0% (0.1%-5.8%) and 17.90% (11.00%-24.3%) (*P* < .0001) in primary cohort, 4.5% (1.20%-7.80%) and 27.3 (18.90-34.90) (*P* < .0001) in the independent validation cohort.

**Conclusions:**

One-year before HCC was a critical period of transitional time when parts of the methylation profile underwent shifting toward HCC like. The nomogram could identify precancerous stage patients with HCC who should be screened for early diagnosis and intervention.

**Implications for practice**

Based on the methylation signature of hepatocellular carcinoma (HCC) trajectory in pseudotime and chronological time, we found 1 year before HCC was a critical transitional period when parts of methylation profile in peripheral blood mononuclear cells underwent changes shifting toward HCC like. The 5-CpG-classifier based on the methylation signature was stratified by clinicopathological risk factors and still kept a clinically and statistically significant predictive factor. The predictive model can be utilized to predict probability of HCC occurrence in the coming 12 months for HBV-infected patients.

## Introduction

Hepatocellular carcinoma (HCC) is the cause of a high rate of morbidity and mortality and represents approximately 90% of all primary liver cancer cases.^1^ More than half of the new liver cancer cases and deaths occur in China.^2,3^ The risk of inducing HCC is increased 10-25 times in patients with surface antigen of hepatitis B virus (HBsAg) positive more than in other non-infected patients.^4^ Early screening of HCC is strongly recommended for HCC surveillance in high-risk chronic hepatitis B (CHB) and patients with liver cirrhosis (LC).^5,6^ Screening patients with HCC who are clinically difficult to diagnose in advance is one of the biggest challenges. Better-performed biomarkers are in urgent need using blood assay.^7^

Aberrant methylation alteration induced by HBV infection contributed to HBV-related HCC and could serve as an excellent tumor marker.^8^ The HCC pathogenesis is a dynamic process encompassing change in systemic molecular, cellular, and physiological states.^9,10^ Our cross-sectional study showed that the methylation changes from CHB to HCC were linked with a key transitional period in HCC development.^11^ When the transitional period occurs and what signature of the period are unknown. To address these important questions at the precise time scale, 17 paired precancerous/HCC peripheral blood mononuclear cells (PBMC) samples and 21 longitudinal PBMC samples from 5 patients were recruited. Experimental design of time-series omics analysis provides an opportunity for investigating important transformation processes and early biomarkers in HCC dynamics.^12,13^ Meanwhile, TimeAx dynamic modeling was applied to overcome patient-specificity, different stages of samples collecting at fixed intervals.^14^

To verify the methylation signature of HCC trajectory, a named 5-CpG-classifier as a biomarker was built to develop and validate an HCC predicting nomogram in a retrospective cohort of HBV-infected patients (*n* = 604). We demonstrated the nomogram utility when applied to screen precancerous stage HCC patients who needed further diagnosis and close observation.

## Methods

### Patients and samples

All samples were obtained from patients treated at the Beijing You’An Hospital, Capital Medical University. The detailed inclusion/exclusion criteria (Supplementary Appendix SE1) and baseline clinicopathological data including demographics, past medical history, habits, blood routine examination, liver and renal function examination, and other data (Supplementary Appendix SE2) were described in Supplementary Data. Patients’ clinicopathological characteristics were gathered when PBMC samples were collected. Whole-blood specimens were collected before the start of standard therapy for the patients. The fresh blood was collected into both citrate and EDTA, and PBMC were isolated by Ficoll-Hypaque density gradient centrifugation within 4 hours and stored at liquid nitrogen.^15^ Informed consent had been obtained from all participants and the study received ethical approval from the Institutional Ethics Committee of the Beijing You’An Hospital.

A total of 22 patients with HCC with their precancerous samples (*n* = 55) and 18 healthy controls samples were gathered from June 13, 2010 to February 24, 2017. The 17 paired precancerous/HCC samples (0-2 years before HCC vs 2 months after HCC) and 21 consecutive samples from 5 patients with 5 time points (3-4 years before HCC, 1-2 years before HCC, 1-year before HCC, and after HCC within 0.5 months) were recruited.

To assess the predictive performance of HCC-associated CpG sites, 604 CHB and LC patients were retrospectively collected from August 15, 2011 to August 28, 2018 by the aid of Beijing Biobank for Hepatitis/AIDS. According to the diagnosis time of CHB or LC, enrolled patients were divided into a primary cohort (*n* = 300, from August 15, 2011 to April 14, 2015) and an independent validation cohort (*n* = 304, from April 15, 2015 to August 28, 2018) to construct and validate an HCC predicting nomogram. The process of excluding incomplete baseline and follow-up information and sample size estimation are shown in Supplementary Figure S6 and Supplementary Appendix SE3.

Follow-up data were obtained by reviewing electronic medical records or telephone communication. We calculated follow-up from the date of PBMC collection to the date of the HCC diagnosis or August 27, 2019, whichever occurred first. HCC occurrence within 1 year was set as an event.

### Pathway analysis

The methods of Infinium HumanMethylation EPIC BeadChip analysis by CapitalBio Technology according to the manufacturers’ guide (Supplementary Appendix SE4) and multiplex bisulfite sequencing (MBS) (Supplementary Appendix SE5) had been published previously.^11,16^ All CpG sites were linked to the gene on the basis of the EPIC annotation file. The “promoter region” of a gene was tightly linked to negative regulation of transcriptional progress, and it was constituted of TSS1500, TSS200, 5ʹUTR, or the 1st exon of gene.^17-20^ We selected the differentially methylated genes whose “promoter region” containing differentially methylated CpG sites (DMCs) to subject to Ingenuity Pathway Analysis (IPA, www.qiagen.com/ingenuity) and Kyoto Encyclopedia of Genes and Genomes (KEGG) to enrich relevant immune signaling and functional pathways analysis.

### Statistical analyses

All analyses were carried out using the R language (version 4.2.2). The cutoff values of quantitative variables were selected applying surv_cutpoint function implemented in “survminer” package or reference ranges of testing items. Infinium EPIC BeadChip arrays were analyzed using the Chip Analysis Methylation Pipeline (ChAMP).^21^ A Bonferroni’s corrected *P* value < .05 was considered as significant. TimeAx was an algorithm based on longitudinal molecular data and predicted HCC of pseudotime positions for all samples.^14,22^ Microarray Significant Profiles (MaSigPro) included 2 steps of regression to search for CpG sites with significant temporal changes.^23^ We analyzed 4 time points and defined a cubic regression model (degree = 3). First, we adjusted this model using the least-squares method to identify differentially expressed CpG sites and selected important ones by applying an error detection rate (*Q* = 0.01). Secondly, a variable selection program was used to search for significant variables for CpG sites, for which we used stepwise regression (step. method=“backward,” alfa = .05). Then, a list of differential CpG sites was generated based on each variable (*R*^2^ > 0.7). The stepwise regression step calculated a *P*-value, if *P* < .05, the line was linear; otherwise, it was parabolic. After maSigPro analysis, a CpG site with a |delta beta| of over 0.3 was considered be differential.

The nomogram was constructed using a least absolute shrinkage and selection operator (LASSO) based Cox-proportional hazard model. Stratified analysis was used to estimate the relationship between the 5-CpG-classifier and the HCC in the coming 1 year within different subsets of the confounding variable. The time-dependent receiver operating characteristic (ROC) curve was obtained using “survivalROC” package. Nomogram was developed using the “rms” package. Area under the ROC curve (AUC) was used to assess predictive capacity. Validation was performed with bias-corrected calibration using bootstrapping method with 1000 resamples. Extension packages, including “ggplot2,” “survminer,” and “pec” were also used.

## Results

### A comparison analysis between pseudotime and chronological time of HCC trajectory

**Figure 1.**
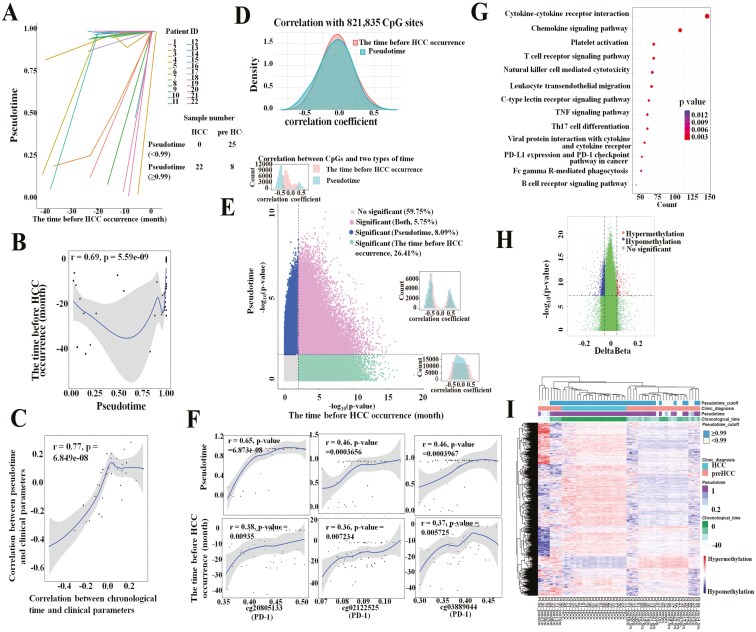
A comparison analysis between pseudotime and chronological time for capturing HCC occurrence dynamics. A comparison between TimeAx model-based pseudotime (*y*-axis) and chronological time (the time before HCC occurrence, *x*-axis) in 22 patients with HCC (A) and in 55 samples (B). Comparison between correlation coefficients (Spearman correlation) for 35 clinical parameters (C) and 821835 CpG sites (D), with either the pseudotime (*y*-axis) or the time before HCC occurrence (*x*-axis), across 55 samples. (E) CpG sites associations (−log_10_ transformed, *P* values based on linear regression) with chronological time (*x*-axis) and pseudotime (*y*-axis), using a *P*-value threshold of .01. Three histograms showing correlation coefficients of associations with 3 types of significant CpG sites between chronological time and pseudotime. (F) Associations of levels of CpG sites located in “promoter region” of the PD-1 with pseudotime (upper) and chronological time (lower). (G) Pathway enrichment analysis of genes whose promoter had CpG sites of significant associations with the pseudotime. (H) Volcano plots displaying 1516 differentially methylated CpG sites between the 8 precancerous stage samples and 22 HCC samples, both their pseudotime no less than 0.99. (I) Heat map of hierarchical clustering of 55 precancerous stages and HCC stages samples by 1516 differentially methylated CpG sites.

The clinical and pathological characteristics of 22 patients with HCC with their precancerous samples collected at several time points are shown in Table 1. The TimeAx model predicted the specific pseudotime of each sample within the original time-series methylation data (Figure 1A, Supplementary Figure S1A). We observed a high correlation between predicted pseudotime and the time before HCC (or chronological time) (*r* = 0.69, Figure 1B, Supplementary Figure S1B) meanwhile, the 2 types of time trajectories both showed a high correlation with 35 clinical parameters (*r* = 0.77, Figure 1C). The pseudotime showed a higher correlation than chronological time with AFP level and age, which were important independent factors associated with HCC (Supplementary Figure S1C and D).

**Table 1.** Clinical and pathological characteristics of 22 patients with HCC with their precancerous samples (*n* = 55).

ID	Sex	Age at HCC diagnosis	Smoking	Alcohol	Time points of PBMC (month)	HCC stage
*The 17 patients with HCC with their paired precancerous/HCC samples (n = 34)*						
Pt.1	Male	50	Heavy	Heavy	−23, 0	BCLC-A
Pt.3	Male	54	No	No	−5, 2	BCLC-A
Pt.6	Male	49	Heavy	Heavy	−14, 0.6	BCLC-A
Pt.7	Female	60	No	No	−15, 1.5	BCLC-A
Pt.8	Male	54	Heavy	No	−16.5, 0.2	BCLC-B
Pt.9	Male	58	Heavy	Low	−23.5, 0.23	BCLC-C
Pt.10	Male	56	No	Quit	−22.5, 0.7	BCLC-A
Pt.12	Male	61	No	No	−6, 0.5	BCLC-A
Pt.13	Female	64	Heavy	No	−7, 1	BCLC-A
Pt.14	Male	52	Heavy	Heavy	−21.5, 0.5	BCLC-A
Pt.16	Female	59	No	No	−18, 0	BCLC-A
Pt.17	Male	52	Heavy	Infrequent	−24, 0	BCLC-C
Pt.18	Male	41	Low	Quit	−13.5, 0	BCLC-C
Pt.19	Male	54	No	No	−21.5, 0.3	BCLC-C
Pt.20	Male	55	Quit	Quit	−12, 0.43	BCLC-B
Pt.21	Male	49	Heavy	No	−8.5. 0	BCLC-A
Pt.22	Female	58	No	No	−10.5, 0.4	BCLC-A
*The 5 patients with HCC with their longitudinal samples (n = 21)*						
Pt.2	Male	58	No	Infrequent	−37, −23, −13, 0.4	BCLC-C
Pt.4	Female	61	No	No	−40, −14.5, −9, 0	BCLC-0
Pt.5	Male	31	Heavy	No	−41, −20.5, −10, 0.2	BCLC-A
Pt.11	Male	55	Quit	Quit	−38, −20, −16, −11, 0.16	BCLC-A
Pt.15	Male	49	No	Infrequent	−24, −20, −12, 0.3	BCLC-B

-: precancerous stage.

Abbreviations: BCLC, Barcelona Clinic Liver Cancer staging system; HCC, hepatocellular carcinoma; PBMC, peripheral blood mononuclear cells; Pt.: patient.

Next, we assessed CpG sites methylation levels as a function of pseudotime. The correlations between CpG sites and pseudotime or chronological time remained in agreement (*r* = 0.75; Figure S1E). Further analysis revealed that associations of CpG methylation levels with chronological time were much stronger (Figure 1D). There were significant associations for more than 26.4% of the CpG sites only with the chronological time, compared with merely 8% and 5.7% for associations with the pseudotime or with both measures, respectively (Figure 1E). Some CpG sites either positively or negatively correlated with the pseudotime and located within “promoter region” (TSS1500, TSS200, 5ʹUTR, and the 1st exon of genes) of immune checkpoint genes (ICGs). These CpG sites had mostly negative regulatory effect on transcription of ICGs including PD-1, CTLA-4, LAG3, TIM3, BTLA, B7H3, VISTA, TIGIT, CD226, and CD96^24,25^ (Figure 1F; Supplementary Figure S2). The methylation alteration of ICGs from precancerous stage to the cancer stage could be captured by pseudotime. The differentially methylated genes only whose “promoter regions” occupied by DMCs were subjected to KEGG pathway enrichment analysis. The results showed that the chemokine signaling pathway, T-cell receptor signaling pathway, PD-L1 expression and PD-1 checkpoint pathway, and Th17 cell differentiation were identified to have strong associations with pseudotime of HCC (Figure 1G).

The minimum pseudotime of the HCC sample was 0.99. According to the pseudotime algorithm, the methylation features of these samples (pseudotime ≥0.99) were set as HCC. We used this cutoff value to divide all samples into 2 groups to analyze their differential signature. Eight precancerous stage samples were divided into the HCC group (Figure 1A). The 1516 DMCs (Bonferroni correction *P* value < .05; |delta beta| > 0.05) between the 8 precancerous stage samples and the HCC sample were analyzed using Limma software (Figure 1H). These DMCs did not show good differentiating ability between precancerous stage and HCC stages (Figure 1I). Meanwhile, there were no DMCs between 8 precancerous stage samples (pseudotime ≥ 0.99) and 25 precancerous stage samples (pseudotime < 0.99) (Supplementary Figure S1F).

Taken together, these results suggested that TimeAx dynamic modeling enabled to capture immune checkpoint signaling signature of the HCC trajectory. However, more specific information and details could be obtained in chronological time of HCC development.

### Chronological time of HCC trajectory analysis revealing 1 year before HCC was a critical period of transitional time

Spearman correlation analysis was performed between the days before or after the HCC diagnosis date and beta values of all CpG sites from 21 consecutive PBMC samples. The analysis revealed a broad signature of DNA methylation that correlated with the pathogenesis of HCC. A genome-wide view of the alteration in DNA methylation of 5193 robust significantly correlated CpG sites (|*r*| > 0.8; *P* < 6.8 × 10^−8^) is shown in Supplementary Figure S3. From TimeAx model, we identified 63.65%, 0.39%, and 30.85% of the 5193 CpG sites significantly associated with the chronological time, pseudotime, or both, respectively (Supplementary Figure S4A). Hierarchical clustering analysis showed clustering of precancerous samples (>12 months) away from HCC samples and precancerous samples (≤12 months) (Supplementary Figure S4B). The features of 5193 CpG sites were verified using 17 paired precancerous/HCC samples. Hierarchical clustering analysis showed clustering of HCC samples and their precancerous stage (≤12 months) away from their precancerous stage (>12 months) (Supplementary Figure S4C). The above results suggested these PBMC DNA methylation profiles of the precancerous stage (≤12 months) represented parallel signatures with early HCC, which provided a critical opportunity in the pathogenesis and diagnosis of early HCC. A comparative IPA canonical pathways analysis revealed that the PD-1/PD-L1 pathway in CD4+ or CD8+ T lymphocytes was activated in precancerous 12 months and HCC stage (Supplementary Figure S4D-I). Overall, the DNA methylation alterations in PBMC showed a signature of ahead of time in immune modulation functions and were consistent with the emerging role of the immune system in HCC.

The 5193 significantly correlated CpG sites were grouped into 9 clusters by MaSigPro based on their similar methylation profiles (Supplementary Figure S5A). The 9 clusters were further grouped by patterns into “up-down” (clusters 1, 2, 6, and 7), “down-up” (clusters 3, 4, 5, 8, and 9), corresponding to changes ranging from precancerous stage to HCC stage. Form linear clusters of 2, 5, 7, and 9, we selected 43 candidate CpG sites based on the most differences between patients with HCC and precancerous stage (≤12 months) and precancerous stage (> 12 months). Using hierarchical clustering, the 43 candidate CpG sites successfully separated the precancerous samples (>12 months) away from all individual HCC samples and precancerous samples (≤12 months) into 2 discrete groups in 21 longitudinal samples (Supplementary Figure S5B) and 17 paired precancerous/HCC samples (Supplementary Figure S5C).

### Construction of the 5-CpG-classifier for prediction of HCC in the coming 1 year

We further validated the predictive value of 43 CpG sites in the HBV-infected patients cohort. A total of 1456 HBV-infected patients whose PBMC samples were available and 633 of them had complete baseline and follow-up information. Apart from other types of cancers, coinfection with HIV and death, finally 604 eligible patients were enrolled in this study (Supplementary Figure S6). In this retrospective study with up to 6.8 years (mean 2.3 years) of follow-up, 67 cases were developing HCC in the coming 1 year.

The detailed clinicopathological characteristics of patients in the primary cohort and independent validation cohort are shown in Table 2. Using MBS data, we applied LASSO-Cox regression to build a prediction 5-CpG-classifier (Supplementary Figure S5D and E), where risk score = 0.4283 × cg13556794 + 2.7307 × cg17375863-0.4972 × cg25174338-0.4144 × cg11823448-0.2322 × cg25334461. Four CpGs were significantly associated with the chronological time, 1 CpG was significantly associated with both pseudotime and chronological time (Supplementary Figure S7). We calculated an HCC risk score for each patient. Setting low-risk groups (score < 0.15) and high-risk groups (score ≥ −0.15) based on the cutoff value selected using the surv-cutpoint function implemented in the application “survminer” software. One-year cumulative hazard of HCC in low- and high-risk groups of patients was 5.0% (0.1%-9.7%) and 12.4% (7.50%-17.0%) in primary cohort (HR = 2.95 (1.46-5.95), *P* = .0026), 5.9% (0.70%-10.80%), and 18.5 (12.7-23.8) in the independent validation cohort (HR = 3.21(1.26-8.19), *P* = .0147) (Table 3, Supplementary Figure S8).

**Table 2.** Clinicopathological characteristics of patients by 5-CpG-classifier assessment set.

	Primary cohort (300)				Independent validation cohort (*n* = 304)		
	Patients (*n*)	Low risk	High risk	Patients (*n*)		Low risk	High risk
Age (years), <53	170	50 (29.41%)	120 (70.59%)	156		48 (30.77%)	108 (69.23%)
Age (years), ≥53	130	39 (30.00%)	91 (70.00%)	148		45 (30.41%)	103 (69.59%)
Sex (male)	228	65 (28.51%)	163 (71.49%)	228		70 (30.70%)	158 (69.30%)
Sex (female)	72	24 (33.33%)	48 (66.67%)	76		23 (30.26%)	53 (69.74%)
History of the first HBsAg+ (years), <9	99	28 (28.28%)	71 (71.72%)	98		37 (37.76%)	61 (62.24%)
History of the first HBsAg + (years), ≥9	201	62 (30.85%)	139 (69.15%)	206		56 (27.18%)	150 (72.82%)
History of CHB, cLC or dLC, (years), <4	192	59 (30.73%)	133 (69.27%)	177		53 (29.94%)	124 (70.06%)
History of CHB, cLC or dLC, (years), ≥4	108	31 (28.70%)	77 (71.30%)	127		40 (31.50%)	87 (68.50%)
HBsAg+/HBeAg+/HBcAb (yes)	97	34 (35.05%)	63 (64.95%)	68		18 (26.47%)	50 (73.53%)
HBsAg+/HBeAg+/HBcAb (no)	150	44 (29.33%)	106 (70.67%)	165		57 (34.55%)	108 (65.45%)
HBsAg+/HBeAb+/HBcAb (yes)	145	42 (28.97%)	103 (71.03%)	160		53 (33.13%)	107 (68.87%)
HBsAg+/HBeAb+/HBcAb (no)	102	36 (35.29%)	66 (64.71%)	73		22 (30.14%)	51 (69.86%)
HBV DNA (IU/mL), <2 × 10^2^	179	56 (31.28%)	123 (68.72%)	196		67 (34.18%)	129 (65.82%)
HBV DNA (IU/mL), 2 × 10^2^ to 2 × 10^5^	92	27 (29.35%)	65 (70.65%)	96		25 (26.04%)	71 (73.96%)
HBV DNA (IU/mL), >2 × 10^5^	29	7 (24.14%)	22 (75.86%)	12		1(8.33%)	11(91.67%)
Smoking (no)	165	48 (29.09%)	117 (70.91%)	184		53 (28.80%)	131 (71.20%)
Smoking (heavy)	135	42 (31.11%)	93(68.89%)	120		40 (33.33%)	80 (66.67%)
Alcohol (no)	158	46 (29.11%)	112 (70.89%)	176		51 (28.98%)	125 (71.02%)
Alcohol (infrequent)	50	14 (28%)	36 (72%)	42		14 (33.33%)	28 (66.67%)
Alcohol (heavy)	92	30 (32.61%)	62 (67.39%)	86		28 (32.56%)	58 (67.44%)
Diagnosis at the time of PBMC collection (CHB, years)	27	12 (44.44%)	15 (55.56%)	11		4 (36.36%)	7 (63.64%)
Diagnosis at the time of PBMC collection, (cLC, years)	36	11 (30.56%)	25 (69.44%)	25		6 (24.00%)	19 (76.00%)
Diagnosis at the time of PBMC collection (dLC, years)	237	67 (28.27%)	170 (71.73%)	268		83 (22.55%)	185 (77.45%)
ALT (U/L), <40	201	64 (31.84%)	137 (68.16%)	221		71 (31.13%)	150 (69.87 %)
ALT (U/L), ≥40	99	25 (25.25%)	74(74.75%)	81		22 (27.16%)	61 (75.31%)
AST (U/L), <40	160	55 (34.37%)	105(65.63%)	184		52 (28.26%)	132 (71.74%)
AST (U/L), ≥40	140	34 (24.29%)	106 (75.71%)	120		41 (34.17%)	79 (65.83%)
TBIL (μmol/L), <21	138	47 (34.06%)	91 (65.94%)	133		38 (28.57%)	95 (71.43%)
TBIL (μmol/L), ≥21	162	42 (25.93%)	120 (74.07%)	171		55 (32.16%)	116 (67.84%)
DBIL, (μmol/L), <7	176	60 (34.09%)	116 (65.91%)	132		37 (28.03%)	95 (71.97%)
DBIL (μmol/L), ≥7	124	29 (23.39%)	95 (76.61%)	172		56 (32.56%)	116 (67.44%)
Total protein (g/L), <65	170	45 (26.47%)	125 (75.53%)	203		62 (30.54%)	141 (69.46%)
Total protein (g/L), ≥65	130	44 (33.85%)	86 (66.15%)	101		31 (30.69%)	70 (69.31%)
Albumin (g/L), <40	220	63 (28.64%)	157 (71.36%)	252		79 (31.35%)	173 (68.65%)
Albumin, (g/L), ≥40	80	26 (32.50%)	54 (67.50%)	52		14 (26.92%)	38 (73.08 %)
γ-GT (U/L), <50	193	58 (30.05%)	135 (69.95%)	193		59 (30.57%)	134 (69.43%)
γ-GT (U/L), ≥50	107	31 (28.97%)	76 (71.03%)	111		34 (30.63%)	77 (69.37%)
ALP (U/L), <125	31	8 (25.81%)	23 (74.19%)	29		10 (34.48%)	19 (65.52%)
ALP (U/L), ≥125	269	81 (30.11%)	188 (69.89%)	275		83 (30.18%)	192 (69.82%)
Hemoglobin (g/L), <130	195	53 (27.18%)	142 (72.82%)	213		66 (30.99%)	147 (69.01%)
Hemoglobin (g/L), ≥130	105	36 (34.29%)	69 (65.71%)	91		27 (29.67%)	64 (70.33%)
MLR, <0.44	237	69 (29.11%)	168 (70.89%)	245		75 (30.61%)	170 (69.39%)
MLR, ≥0.44	63	20 (31.75%)	43 (71.67%)	59		18 (30.51%)	41 (69.49%)
NLR, <1.56	108	30 (27.78%)	78 (72.22%)	93		34 (36.56%)	59 (63.44%)
NLR, ≥1.56	192	59 (30.73%)	133 (69.27%)	211		59 (27.96%)	152 (72.04%)
PLR, <53.5	83	23 (27.71%)	63 (72.29%)	57		17 (29.82%)	40 (70.18%)
PLR, ≥53.5	217	66 (30.41%)	151 (69.59%)	247		76 (30.77%)	171 (69.23%)
AFP (ng/mL), <7	203	61 (30.05%)	142 (69.95%)	221		67 (30.32%)	154 (69.68%)
AFP (ng/mL), ≥7	97	28 (28.87%)	69 (71.13%)	83		26 (31.32%)	57 (68.67%)

Abbreviations: AFP, alpha-fetoprotein; ALP, alkaline phosphatase; ALT, alanine aminotransferase; AST, aspartate aminotransferase; cLC: compensated liver cirrhosis; DBIL, direct bilirubin; dLC: decompensated liver cirrhosis; MLR, the monocyte to lymphocyte ratio; NLR, the neutrophil to lymphocyte ratio; PLR, the platelet to lymphocyte ratio; TBIL, total bilirubin; γ-GT, γ-glutamyltranspeptidase

**Table 3.** Univariate and multivariable Cox regression analysis of association of 5-CpG-classifier, clinicopathological characteristics with HCC occurrence.

	Primary cohort (*n* = 300)		Independent validation cohort (*n* = 304)	
	HR (95% CI)	*P* value	HR (95% CI)	*P* value
*Univariate Cox regression analysis*				
Age (years), ≥53 vs<53	1.82 (1.11-2.97)	.0161	1.29 (0.69-2.40)	.424
Sex (female vs male)	0.47 (0.23-0.94)	.0331	0.32 (0.11-0.90)	.0312
History of the first HBsAg+, (years), ≥9 vs <9	2.71 (1.38-5.31)	.00364	1.9 (0.87-4.12)	.104
History of CHB, LC or DLC, (years), ≥4 vs <4	1.29 (0.8-2.08)	.303	0.98 (0.53-1.86)	.982
HBsAg+/HBeAg+/HBcAb, (yes vs no)	1.17 (0.71-1.93)	.531	1.41 (0.74-2.70)	.297
HBsAg+/HBeAb+/HBcAb, (yes vs no)	0.92 (0.56-1.51)	.74	0.78 (0.41-1.49)	.452
HBV DNA (IU/mL), 2 × 10^2^ to 2 × 10^5^ vs < 2 × 10^2^	1.30 (0.78-2.17)	.996	1.36 (0.70-2.62)	.366
HBV DNA (IU/mL), >2 × 10^5^ vs <2 × 10^2^	1.06 (0.38-2.97)	.116	1.57 (0.36-6.38)	.577
Smoking, heavy vs no	1.43 (0.70-2.31)	.143	1.37 (0.73-2.54)	.323
Alcohol, infrequent vs no	1.18 (0.60-2.32)	.639	0.93 (0.35-2.47)	.888
Alcohol, heavy vs no	1.23 (0.72-2.10)	.452	1.32 (0.67-2.60)	.419
Diagnosis at the time of PBMC collection, LC vs CHB	1.49 (0.30-7.37)	.627	0.35 (0.05-2.52)	.300
Diagnosis at the time of PBMC collection, DLC vs CHB	1.91 (0.47-7.80)	.370	0.65 (0.16-2.72)	.558
ALT (U/L), ≥40 vs <40	1.07 (0.62-1.77)	.863	0.82 (0.39-1.72)	.593
AST (U/L), ≥40 vs <40	0.73 (0.44-1.21)	.222	0.52 (0.25-1.07)	.0746
TBIL (μmol/L), ≥21 vs <21	0.80 (0.49-1.29)	.366	0.66(0.35-1.23)	.192
DBIL (μmol/L), ≥7 vs <7	1.30 (0.81-2.11)	.277	1.04 (0.55-1.94)	.912
Total protein (g/L), ≥65 vs <65	1.21 (0.74-1.96)	.446	1.48 (0.79-2.76)	.224
Albumin (g/L), ≥40 vs <40	1.26 (0.74-2.17)	.396	1.36 (0.65-2.86)	.413
γ-GT (U/L), ≥50 vs<50	1.01 (0.62-1.67)	.955	0.94 (0.49-1.81)	.864
ALP (U/L), ≥125 vs<125	3.79 (0.93-15.51)	.0631	4.57 (0.63-33.28)	.133
Hemoglobin (g/L), ≥130 vs <130	1.27 (0.78-2.08)	.339	1.43 (0.75-2.70)	.278
MLR, ≥0.44 vs <0.44	1.05 (0.58-1.89)	.863	0.89 (0.38-1.96)	.737
NLR, ≥1.56 vs <1.56	1.52 (0.87-2.63)	.138	1.78 (0.82-3.85)	.147
PLR, ≥53.5 vs <53.5	0.54 (0.33-0.88)	.0145	0.44 (0.23-0.84)	.0129
AFP (ng/mL), ≥7 vs <7	2.02 (1.24-3.29)	.00448	2.44 (1.31-4.58)	.00523
Five-CpG-classifier, −0.15 vs <−0.15	2.95 (1.46-5.95)	.00256	3.21 (1.26-8.19)	.0147
*Multivariable Cox regression analysis*				
Age (years), ≥53 vs <53	2.10(1.28-3.45)	.0035	1.40 (0.75-2.62)	.2914
Sex (female vs male)	0.43 (0.21-0.88)	.0208	0.33 (0.12-0.96)	.0421
History of the first HBsAg+, (years), ≥9 vs <9	2.90(1.47-5.70)	.0020	2.16 (0.98-4.76)	.0554
AFP (ng/mL), ≥7 vs <7	1.93 (1.17-3.18)	.0097	2.35 (1.23-4.48)	.0092
PLR, ≥53.5 vs <53.5	0.63 (0.37-1.07)	.00887	0.57 (0.29-1.15)	.1180
Five-CpG-classifier, ≥−0.15 vs <−0.15	2.69(1.44-5.44)	.0058	3.08 (1.20-7.90)	.0195

Abbreviations: AFP, alpha-fetoprotein; ALP, alkaline phosphatase; ALT, alanine aminotransferase; AST, aspartate aminotransferase; DBIL, direct bilirubin; MLR, the monocyte to lymphocyte ratio, NLR, the neutrophil to lymphocyte ratio; PLR, the platelet to lymphocyte ratio; TBIL, total bilirubin; γ-GT, γ-glutamyltranspeptidase.

The univariate and multivariable analysis by clinicopathological characteristics and 5-CpG-classifier in the 2 cohorts are shown in Table 3. After multivariable adjustment by clinicopathological variables, the 5-CpG-classifier remained a significant and independent factor associated with HCC occurrence in the primary cohort (HR = 2.69 (1.44-5.44), *P* = .0058), as well as in the independent validation set (HR = 3.08 (1.20-7.90), *P* = .0195) (Table 3).

**Figure 2.**
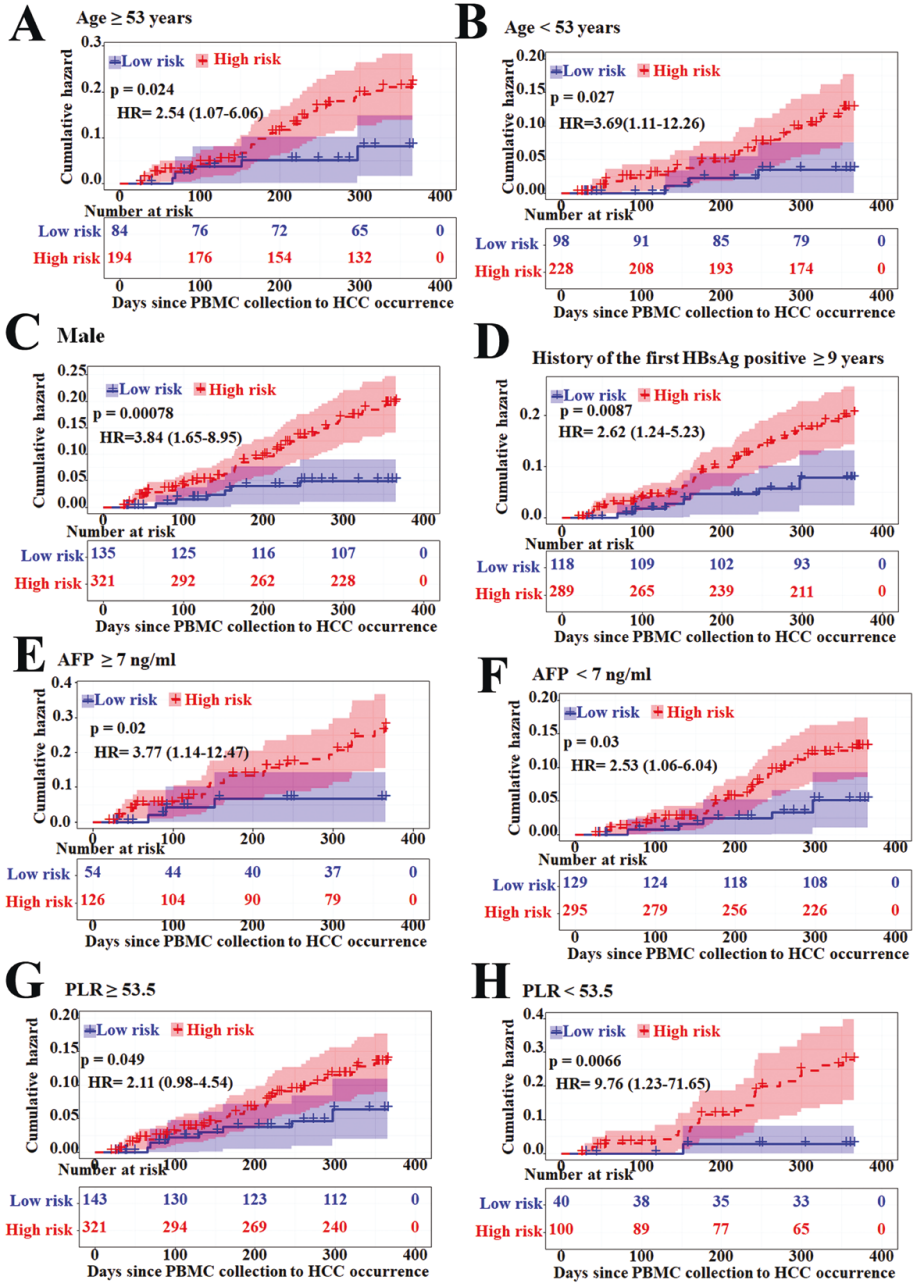
Kaplan-Meier survival analysis for all 604 patients according to the 5-CpG-classifier stratified by clinicopathological risk factors. (A, B) Age; (C) male; (D) history of the first HBsAg positive ≥9 years; (E, F) alpha-fetoprotein level; (G, H) PLR (the platelet to lymphocyte ratio). *P* values were calculated using a log-rank test and hazard ratios using a univariate Cox regression analysis. Abbreviation: HR: hazard ratio.

When stratified by clinicopathological risk factors, the 5-CpG-classifier was still a clinically and statistically significant prediction model (Figure 2; Supplementary Table S1), except for females and history of the first HBsAg positive <9 years (Supplementary Figure S9). The distribution of clinicopathological characteristics did not vary significantly between the high-risk and low-risk groups both in the primary cohort and independent validation cohort. These results clearly indicated that the association of 5-CpG-classifier with prediction of HCC was not affected by clinicopathological risk factors.

### Development and validation of an early HCC predicting nomogram based on 5-CpG-classifier

**Figure 3.**
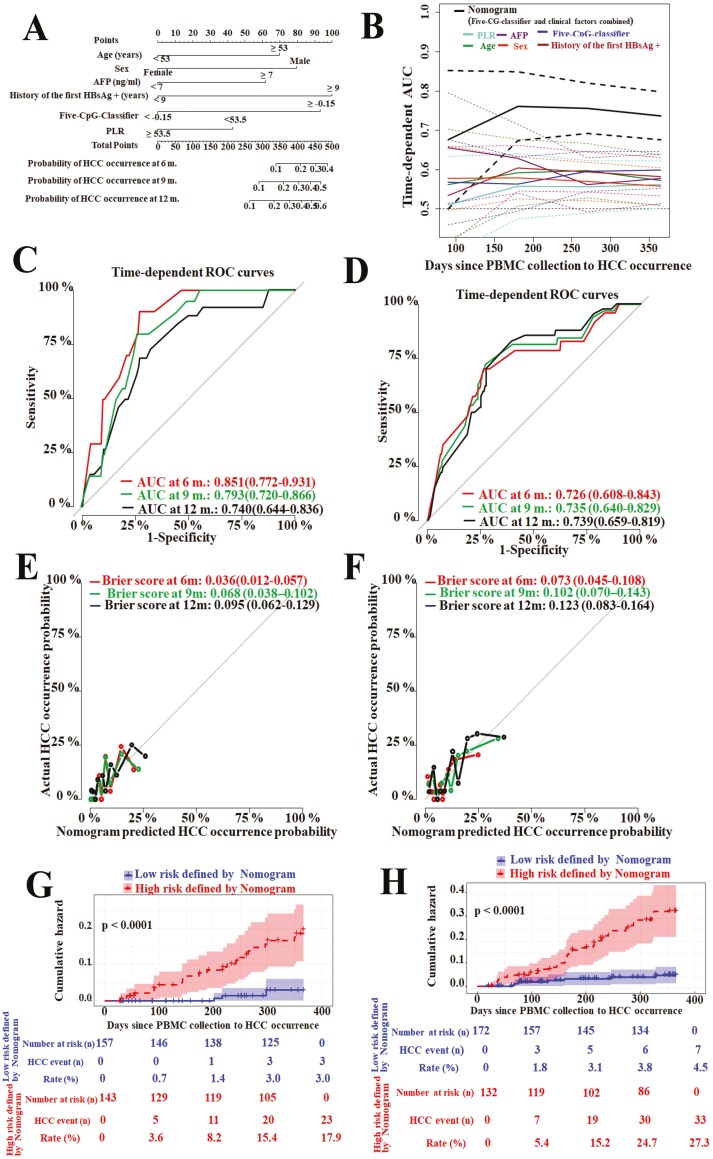
The nomogram predicted the risk of HCC occurrence in the coming 12 months. (A) The nomograms for predicting the proportion of patients with HCC occurrence were created based on 6 independent prognostics factors including age, sex, AFP, 5-CpG-classifier, history of the first HBsAg positive, and PLR (the platelet to lymphocyte ratio). (B) Time-dependent area under the curves with 95% CIs of nomogram and 6 independent prognostics factors for HCC prediction over time since PBMC collection to HCC occurrence (90-365 days). Time-dependent ROC curves by nomogram for HCC occurrence probability at 6-, 9-, 12- months since PBMC collection in primary cohort (C) and independent validation cohort (D). Calibration curves of nomogram in terms of agreement between predicted and actual HCC occurrence probability at 6-, 9-, 12- months since PBMC collection in primary cohort (E) and independent validation cohort (F). The cumulative hazard with 95% CIs of HCC was shown using Kaplan-Meier curves for the low-risk group and high-risk group defined by nomogram in the primary cohort (G) and independent validation cohort (H). *P* values were determined using log-rank testing.

A nomogram was constructed to provide the clinician with a quantitative method predicting a patient’s probability of HCC within coming 1 year (Figure 3A). The coefficients of 6 factors of the nomogram are listed in Supplementary Table S2. The 5-CpG-classifier had outstanding predictive accuracy and a major contribution to predicting performance (Figure 3B). The time-dependent ROC curves showed AUC values for the prediction of HCC at 6-, 9-, and 12- months since PBMC collection in the primary cohort (Figure 3C) and in the independent validation cohort (Figure 3D). Calibration plots showed that the probability predicted by the nomogram agreed well with actual HCC occurrence probability in the primary cohort (Figure 3E), and in the independent validation cohort (Figure 3F). Using this nomogram, 1-year cumulative hazard of HCC in low- and high-risk groups were 3.0 % (0.1%-5.8%) and 17.90% (11.00%-24.3%) in primary cohort (*P* < .0001) (Figure 3G), 4.5% (1.20%-7.80%) and 27.3 (18.90-34.90) in independent validation cohort (*P* < .0001) (Figure 3H). These results revealed that the early HCC-predicting nomogram helped identify the precancerous stage of patients with HCC.

## Discussion

Our work differed from previous HCC early detection studies which focused on biomarkers that only appeared during the HCC period. Our work combined systematic analysis of longitudinal DNA methylation data to investigate similar methylation signatures both in precancerous and HCC stages and validated them as markers for early HCC predicting. Such an assumption reduced the limitation of many previous studies when precancerous stage patients were unnoticed. In fact, they were real high-risk patients and should be screened for early diagnosis and intervention.

To the best of our knowledge, this is the first report discussing the methylation changes of HCC occurrence on pseudotime and chronological time. Based on TimeAx dynamic modeling, pseudotime of precancerous/HCC stage samples capturing methylation alteration of ICGs indicated that PD-L1 expression and PD-1 checkpoint pathway^26^ were involved in HCC dynamics. TimeAx dynamic modeling was not the same as with differences analysis method. More than half of 350 differentially methylated CpG sites^11^ had no significant associations with pseudotime or/and chronological time (Supplementary Figure S5A). The chronological time of the HCC trajectory showed that 1 year before HCC was a critical transitional period when parts of the methylation profile changed ahead of time shifting toward HCC like. More importantly, the 5193 robust significantly correlated CpG sites offered a critical opportunity to investigate pathogenesis and early prediction.

An aberrant methylation alteration is one of the main responsibilities for vast majority of cancers.^27^ These accumulations continuously took place at low levels in normal-appearing tissues until cancer occurrence, constituting cancer risk.^28^ Accordingly, caner-related aberrant methylation characteristic was detected both from non-tumor biopsies^29^ and precancerous samples.^30^ The signature of this methylation biomarker offered an opportunity to improve cancer diagnosis spatially and temporally. The preneoplastic methylation changes could be observed in benign tissues, that was to say the concept of an epigenetic field cancerization, which could explain the multifocality of colon,^31^ bladder,^32^ and prostate.^33^ The abnormal changes of specific DNA methylation locus even appeared in the regions spatially remote (> 1 cm) from prostate tumor-bearing areas.^33^ These findings made it possible to detect patients with higher-risk prostate cancer with histologically negative biopsies^29,33^ even in urine,^34^ thereby decreasing the need for repeated prostate biopsies. A similar study had also been confirmed in the diagnosis of renal cancer.^35^

Meanwhile, alteration of blood DNA methylation occurred several years in advance could be used to predict the risk of future neoplastic transformation,^30,36^ especially chronic inflammation-associated cancers such as gastric neoplasms and HCC.^37^ A total of 140 risk CpG sites in blood presented high sensitivity and specificity to detect pre-invasive neoplasia (AUC = 0.93, 95% CI, 0.86-1) and cervical cancer (AUC = 1) occurred within future 3 years.^30^ A large-scale prospective cohort (*n* = 2324) with follow up of up to 28 years (mean 15 years) selected 126 CpG sites for lymphatic-hematopoietic cancers (*n* = 41), 396 for solid cancers, and 414 for overall cancers as risk factors that constituted a precision medicine tool for the early identification of cancers.^36^

The 5-CpG-classifier, an epigenetic risk marker, was considered to reflect exposure to a chronic inflammation environment, and a history of HBsAg positive reflecting HBV infection was associated with the prediction of HCC. HBsAg-positive individuals in the community population were at high risk of HCC, with 0.105% (4/3793) cumulative incidence rate following up 6-8 months.^6^ In a systematic review and meta-analysis of 12,541 CHB patients, the OR for HCC of HBsAg levels (≥100 IU/mL vs <100 IU/mL) was 4.99 (95% CI, 3.01-8.29), and at the 1000 IU/mL threshold, the OR was 2.46 (95% CI, 2.15-2.83).^38^ In this study, the history of HBsAg first-time positive, rather than the history of CHB or LC, was associated with an increased risk of HCC. Both CHB and LC were high-risk factors for HCC, but that did not mean each patient had an equivalent risk.^39^ Addressing the heterogeneity of HBV-related liver disease, an effective strategy of risk stratification identified patients at a high risk of developing HCC.

Methylation biomarkers are promising directions for HCC diagnosis in HBV-infected patients. Meanwhile, methylation biomarkers have been recommended as a tool for tumor detection by many expert consensuses in China.^40,41^ At the same time, many methylation biomarker kits are brought into China National Medical Insurance System for tumor detection. The use of the 5-CpG-classifier could improve the performance of our model. To accelerate the implementation of clinical applications, we will upgrade the detection method of CpG sites by using a digital PCR technology, which is shorter, faster, and more suitable for clinical requirements.

Our study had several limitations. Some specific candidate CpG sites biomarkers might be abandoned because of compatibility with requirements of CpG free in multiplex PCR primer design. It was worth making more efforts in the design of multiplex PCR primer pairs for as many as possible candidate CpG sites. The cumulative incidence of HCC among HBV-infected patients in this study had not been calculated from the time of the initial visit. The HCC nomogram was more suitable for screening precancerous patients with HCC in high-risk populations. A large cohort external validation in a prospective setting would be further required to clarify our 5-CpG-classifier and the predicting nomogram in multicenter clinical studies.

## Conclusions

In summary, our findings showed that methylation changes of HCC on pseudotime and chronological time. One year before HCC was a critical period of transitional time when parts of the methylation profile underwent changes shifting toward HCC like. Moreover, we developed and validated an HCC-predicting nomogram based on 5-CpG-classifier to predict the HCC risk of HBV-infected patients in the coming 1 year. Therefore, the nomogram led to early diagnosis and close observation for patients with precancerous HCC.

## Supplementary Material

oyae292_suppl_Supplementary_Appendix

oyae292_suppl_Supplementary_Figures

oyae292_suppl_Supplementary_Tables

## Data Availability

The data underlying this article will be shared on reasonable request to the corresponding author.
