# Supplementary material for: The methylation signature of hepatocellular carcinoma trajectory based on pseudotime and chronological time for predicting precancerous patients
Source: Oncologist. 2024 Nov 26;30(8):oyae292. doi: 10.1093/oncolo/oyae292 (PMC12395135; doi:10.1093/oncolo/oyae292)
Supplement: oyae292_suppl_Supplementary_Appendix [file oyae292_suppl_supplementary_appendix.docx]

**Appendix E1: Inclusion and exclusion criteria**

The inclusion and exclusion criteria were applied in this study, as follows:

The inclusion criterion of CHB was that hepatitis B surface antigen (HBsAg) seropositive status lasted at 6 months or beyond according to the Asia-Pacific clinical practice guidelines on the management of hepatitis B: a 2015 update. Diagnosis of hepatitis B-related liver cirrhosis (LC) patients was based on a combination of clinical, laboratory, imaging features, and liver biopsies according to the guideline of prevention and treatment for chronic hepatitis B (2010 Version). Diagnosis of HCC was based on the radiological/histological criteria according to 2012 EASL clinical practice guidelines: EASL Clinical Practice Guidelines: Management of hepatocellular carcinoma. HCC was classified based on the Barcelona Clinic Liver Cancer (BCLC) staging system. Exclusion criteria were as follows: less than 18 years old; other types of cancers; co-infection with human immunodeficiency virus, hepatitis C virus, or hepatitis D virus; coexistence of liver injury caused by drug intake, and autoimmune hepatitis; pregnancy; and lactation.

**Appendix E2: Demographic and clinicopathological data**

In this study, demographic and baseline clinicopathological data were as follows: (1) demographic data: age, sex; (2) past history: the history of the first HBsAg positive, the history of CHB, LC or DLC, the history of antiviral, (3) habits: the history smoking and alcohol drinking; (4) blood routine examination: white blood cell, neutrophil, lymphocyte, monocyte, hemoglobin, platelet; (5) liver and renal function examination: Child–Pugh class; alanine aminotransferase (ALT), aspartate aminotransferase (AST), total bilirubin (TBIL), direct bilirubin (DBIL), total protein (TP), albumin, globulin, γ-glutamyl transpeptidase (γ-GT), alkaline phosphatase (ALP), prealbumin, total bile acid (TBA), (6) other indicators: alpha fetoprotein (AFP) and viral load.

**Appendix E3: The Sample Size estimation**

In our study, according to the diagnosis time of HBVLD, enrolled patients were divided into primary cohort (n=300, from August 15th, 2011 to April 14th, 2015) and independent validation cohort (n=304, from April 15th, 2015 to August 28th, 2018) to construct and validate an HCC screening nomogram. The minimum sample size for constructing the nomogram was 270 according to calculations carried out in PASS version 11.0.7 (85% power at a 0.05 significance level to detect a hazard ratio of HCC-associated CpG sites combination equal to 3) The sample size was adjusted since a multiple regression of the HCC-associated CpG sites combination on the other covariates in the Cox regression is expected to have an R-Squared of 0.15. In our study, the data from 300 patients from primary cohort were used to construct the nomogram, which was able to meet the sample size requirement.

For the validation cohorts, we applied MedCalc to calculate the required sample size for the comparison of AUC of HCC occurrence within one year (about 0.75) with a null hypothesis value (0.6). The Type I error (α-level, significance, two-sided) was select 0.05 and Type II error (β-level) was select 0.10 (power is 90%). Ratio of sample sizes in negative/positive groups was 0.111. Finally, 31 cases were required in the positive group and 273 in the negative group, giving a total of 304 cases.

**Appendix E4: Infinium** **HumanMethylation EPIC BeadChip analysis**

PBMC DNA was treated with bisulfite converting unmethylated cytosine residues to uracil (ZYMO Research, Irvine, USA), and tested using Infinium HumanMethylation EPIC BeadChip array (Illumina, Inc., San Diego, CA) by CapitalBio Technology according the manufactures’ guide. In brief, converted genome DNA was isothermally amplified, enzymatically fragmented, precipitated, denatured, and hybridized on the BeadChips. Then BeadChips were washed, extended with biotin modified ddCTPs, ddGTP or DNP modified ddATP, ddTTP prior to scanning with iScan system (Illumina, Inc., San Diego, CA). In consequence, 866,836 CpG sites were measured on the EPIC arrays. The DNA methylation raw data was preprocessed using Chip Analysis Methylation Pipeline (ChAMP). It mainly included loading raw intensity data (IDAT) files, filtering out probes, quality check, normalization, and batch effect correction. The probes were removed out as follows: detection *P* value > 0.01, a beadcount < 3 in at least 5% of samples, containing single nucleotide polymorphisms (SNPs) aligning to multiple locations, locating on X, Y chromosome as well as non-CpG.

**Appendix E5: Multiplex bisulfite sequencing (MBS)**

The methylation level of 43 HCC-associated CpG sites in 604 PBMC samples were measured using MBS. PBMC DNA was converted (ZYMO Research, Irvine, USA) and measured the concentration of the single strand DNA using a Qubit 2.0 (Thermo, Carlsbad, CA, USA). A panel of 43 paired ‘CpG-free’ primers was designed for targeting to all candidate CpGs. Library preparation was performed by nest-PCR, the bisulfite converted DNA served as template binding by panel of 43 primer pairs in the first round PCR reaction. The first round PCR amplicon served as template, universal P7 primer with barcode and universal P5 primer with barcode served as forward and reverse primers respectively in the second PCR reaction. Then, the final PCR amplicon was purified using AMPure XP beads. The libraries were then quantified and pooled. Paired-end sequencing of the library was performed on the HiSeq XTen sequencers (Illumina, San Diego, CA). MBS raw data was processed by Sangon Biotech. Cutadapt (v 1.2.1) was applied to remove adaptor sequence; PRINSEQ-lite (v 0.20.3) was applied to remove low quality bases from reads 3’ to 5’ (Q < 20); and Bismark (version v0.22.1) was applied for CpG sites detection with default parameters.
