# Supplementary material for: The methylation signature of hepatocellular carcinoma trajectory based on pseudotime and chronological time for predicting precancerous patients
Source: Oncologist. 2024 Nov 26;30(8):oyae292. doi: 10.1093/oncolo/oyae292 (PMC12395135; doi:10.1093/oncolo/oyae292)
Supplement: oyae292_suppl_Supplementary_Figures [file oyae292_suppl_supplementary_figures.docx]

**Supplementary Figures**


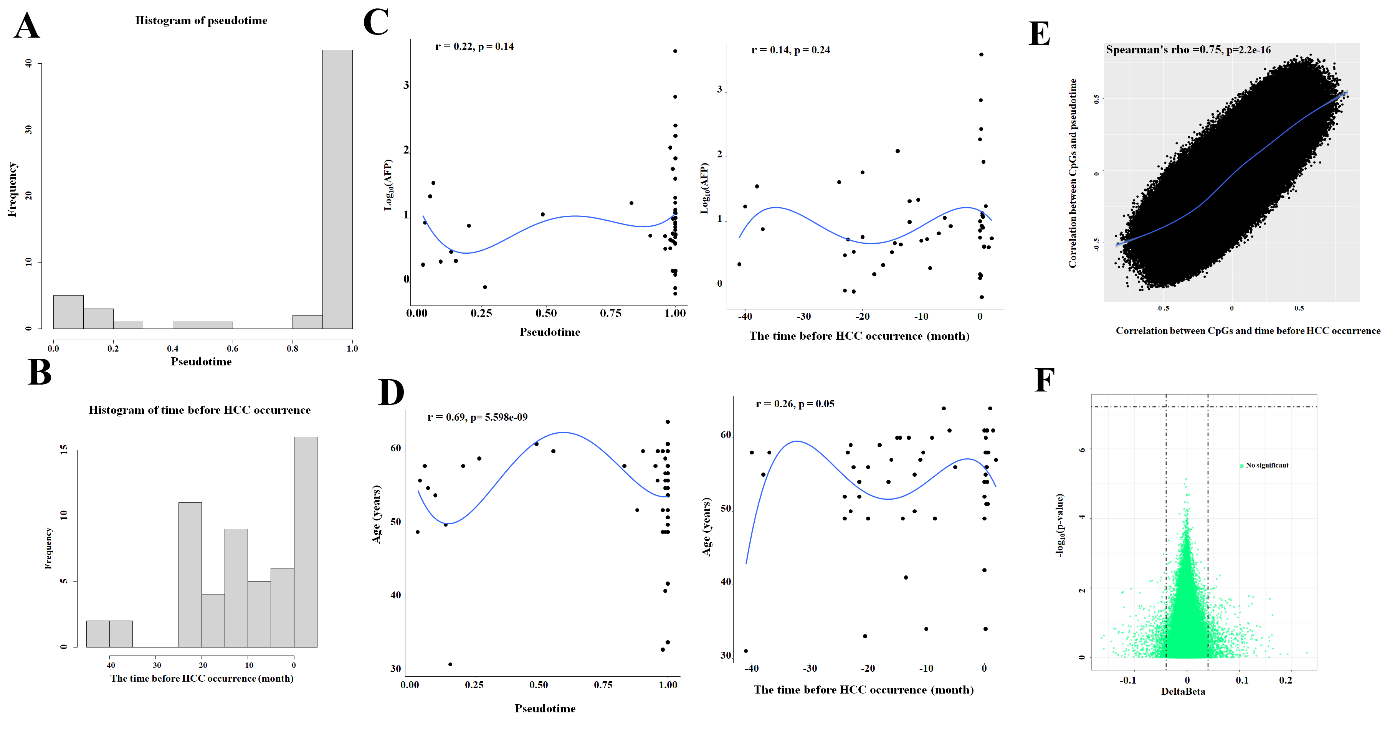


**Figure S1. TimeAx model-based pseudotime and chronological time of 55 samples.** (A) Histogram of time before HCC occurrence; (B) Histogram of pseudotime; (C) A comparison analysis between alpha-fetoprotein (AFP) between pseudotime and chronological time; (D) A comparison analysis between age between pseudotime and chronological time. (E) Comparison between correlation coefficients (Spearman correlation) for all 821835 CpG sites, with either the pseudotime or the time before HCC occurrence; (F) Volcano plots displaying no differentially methylated CpG sites between the eight precancerous stage samples (pseudotime ≥ 0.99) and 25 precancerous stage samples (pseudotime < 0.99).


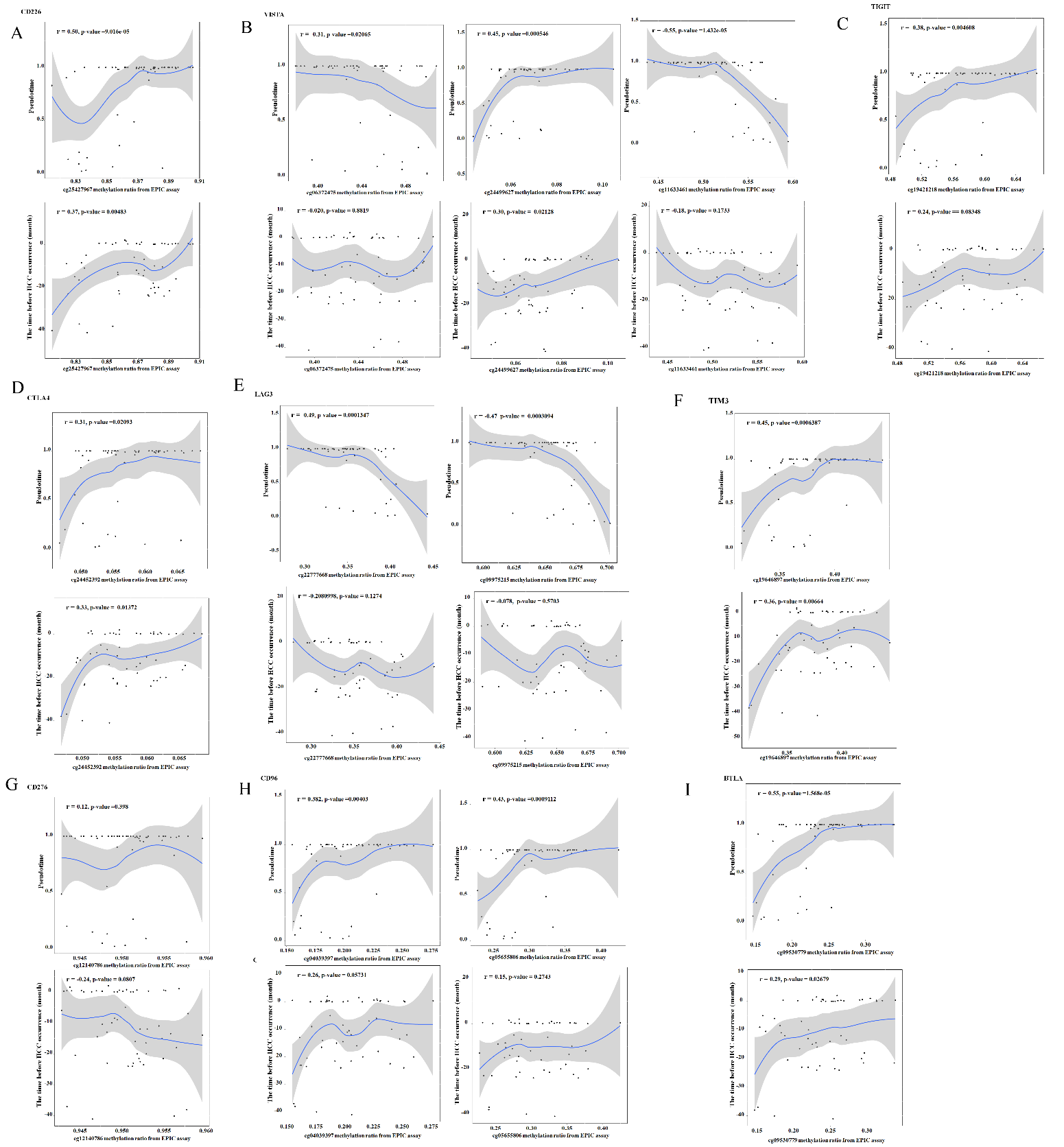


**Figure S2. Correlation of CpG sites located within** **‘promoter region’ of immune checkpoint genes (ICGs) with the pseudotime** (**A**) CD226 (cg25427967), (**B**) V-domain Ig suppressor of T cell activation (VISTA) (cg06372475, cg24499627 and cg11633461), (**C**) T cell immunoreceptor with Ig and ITIM domains (TIGIT) (cg19421218), (**D**) Cytotoxic T-lymphocyte associated protein 4 (CTLA 4) (cg24452392), (**E**) Lymphocyte activating 3 (LAG3) (cg22777668 and g09975215), (**F**) T-cell immunoglobulin mucin family member 3 (TIM3) (cg19646897), (**G**) CD276 (cg12140786), (**H**) CD96 (cg04039397, cg05655806), (**I**) B- And T-Lymphocyte Attenuator (BTLA) (cg09530779)


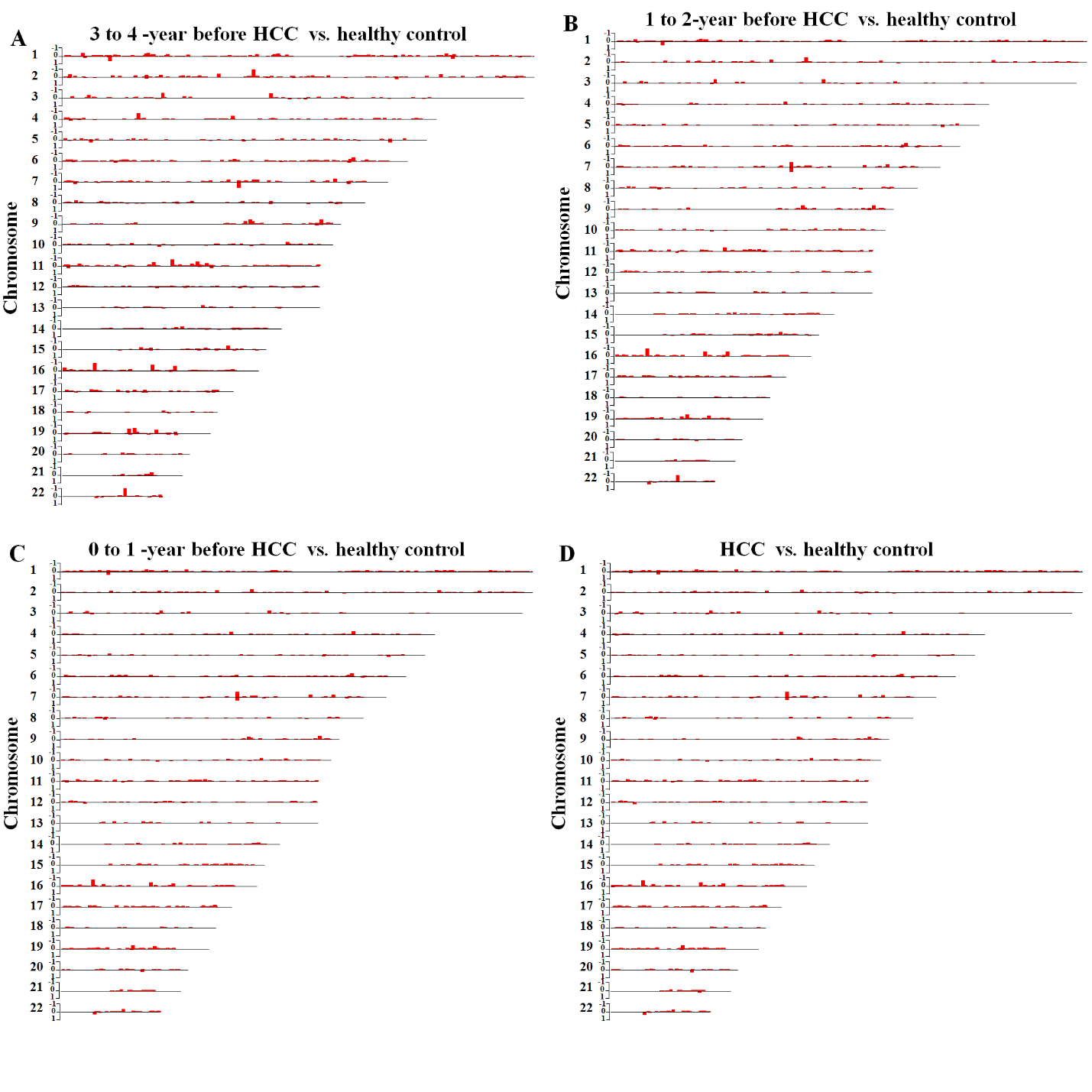
**Figure S3.** Human autosome ideograms showing 5193 robust significantly correlated CpG sites in three-four years before HCC (A), one-two years before HCC (B), one year before HCC (C), and after HCC within 0.5 months (D) compared to healthy control.


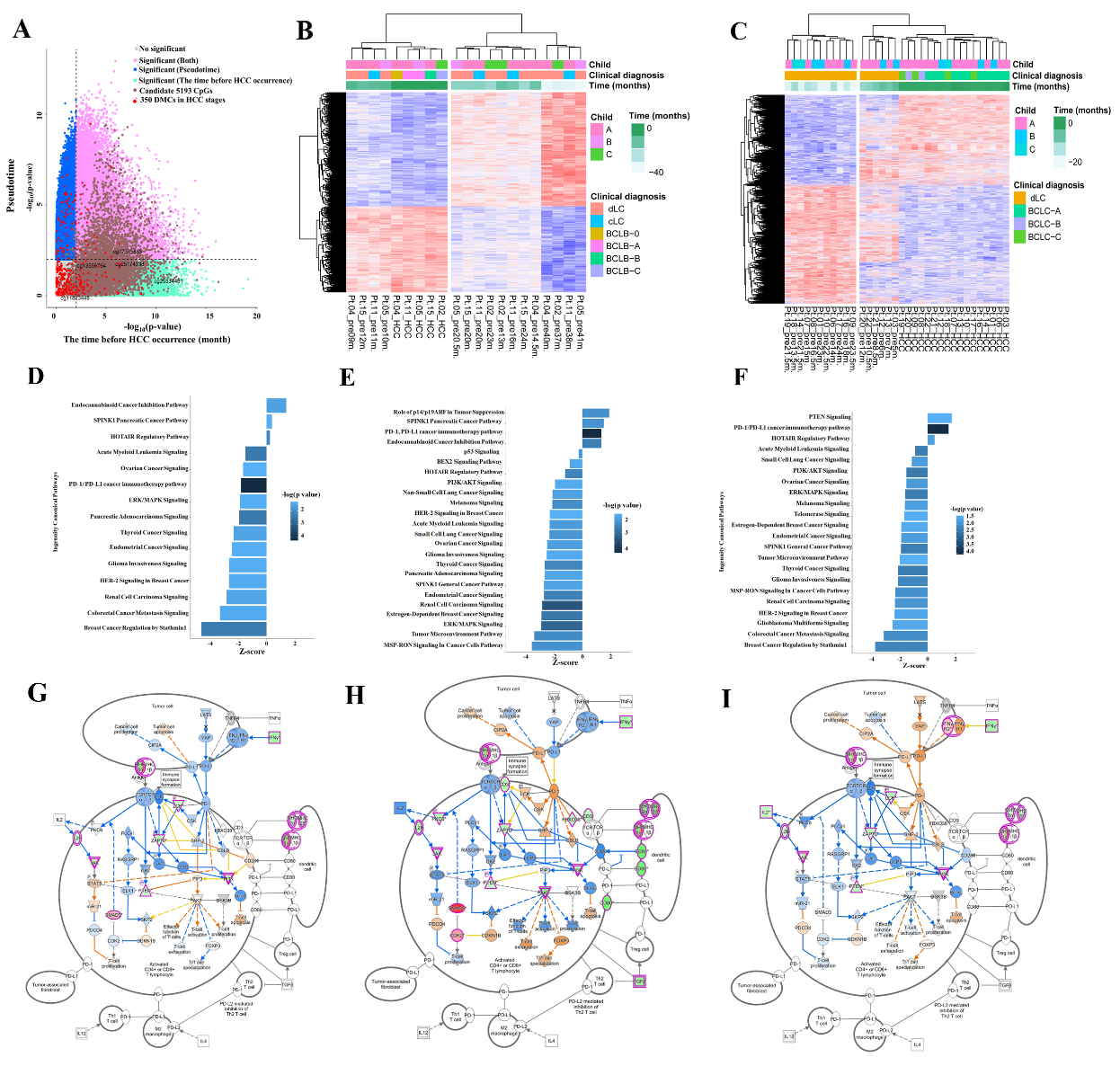


**Figure S4. Correlation between quantitative distribution of specific CG sites methylation levels and pathogenesis of HCC.** (A) The 5193 robust significantly correlated CpG sites were evaluated using TimeAx model. The brown dots showing the 5193 CpG sites of associations (-log_10_ transformed, p values based on linear regression) with chronological time (x-axis) and pseudotime (y-axis), using a p value threshold of 0.01. The red dots showing 350 differentially methylated CpG sites in HCC stage 1, 2, 3 and 4 compared to healthy control that reported in our previous study.^1^ (B) Heat map of hierarchical clustering 21 longitudinal PBMC samples from 5 patients using Spearman correlation of DNA methylation beta values of the 5193 robust significantly correlated CpG sites (r > 0.8; r < − 0.8; p < 6.8×10^−8^). (C) Heat map of hierarchical clustering 17 paired precancerous/HCC samples using DNA methylation beta values of the 5193 robust significantly correlated CG sites. IPA-predicted most affected bio-functions by differentially methylated genes in precancerous sage (> 12 months) (D), precancerous stage (≤ 12 months) (E) and HCC stage (F). The default IPA Z-score settings for activation (+1.96σ) and inhibition (-1.96σ) were used. The p-value, calculated with the Fischer's exact test, (p-value ≤ 0.05 (i.e., −log_10_ ≥ 1.3) was significant. A comparative IPA canonical pathways analysis showing activation status of PD-1/PD-L1 pathway in CD4+ or CD8+ T lymphocyte in precancerous sage (> 12 months) (G), precancerous stage (≤ 12 months) (H) and HCC stage (I). The blue represented inactive status, orange represented active status. IPA: Ingenuity Pathway Analysis.


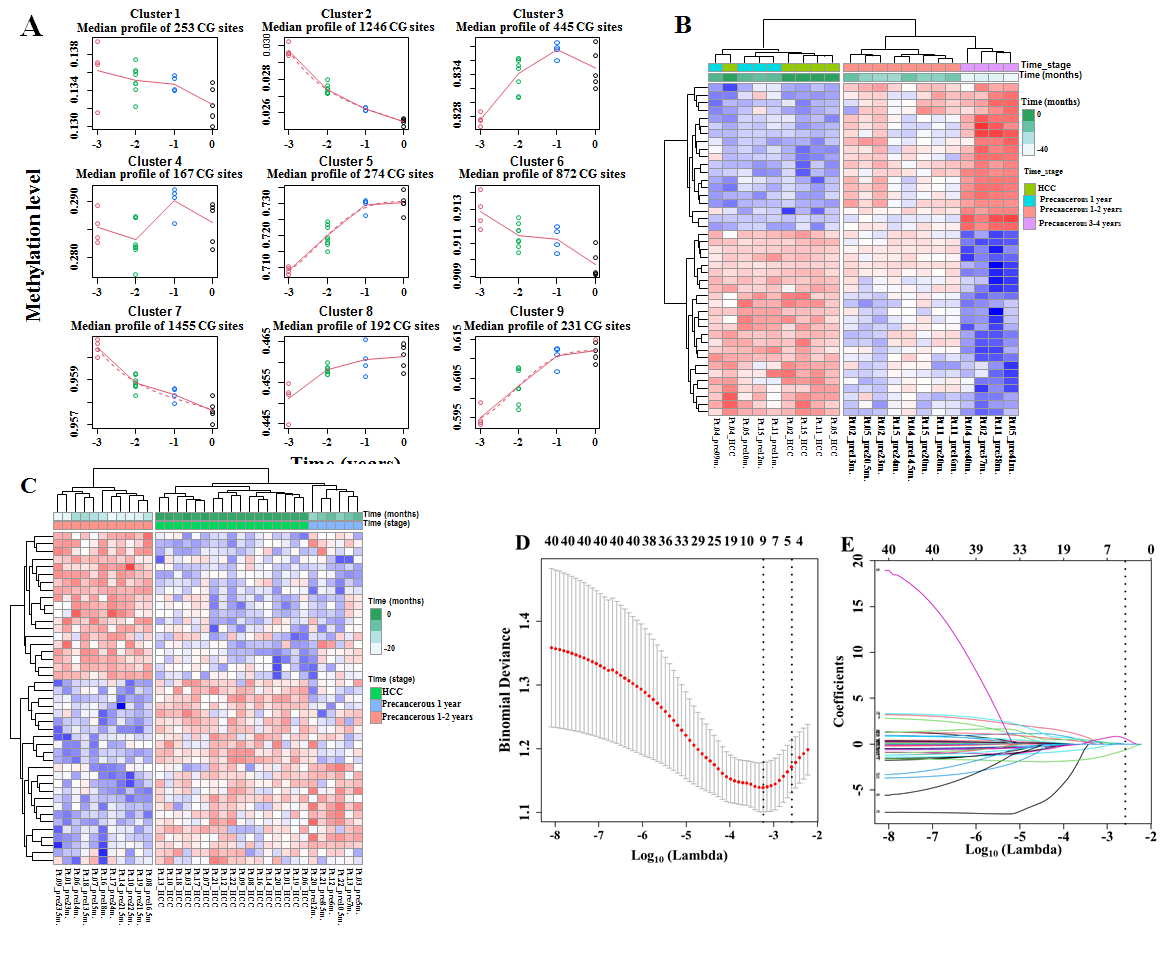
**Figure S5. Construction of the five-CpG-classifier for prediction of HCC occurrence.** (A) Microarray Significant Profiles (maSigPro) was used to analyze profiles of methylation level of CpG sites. The 5193 significantly correlated CpG sites were grouped into nine clusters. Clustering method for data portioning was K-means. Dots showed actual methylation level. Solid lines had been drawn joining the average beta value of CpG sites methylation level at each time point for HCC pathogenesis. Dotted lines displayed fitted curves. “-” represented precancerous stage. (B) Heat map of hierarchical clustering 21 longitudinal PBMC samples from five patients using DNA methylation beta values of the 43 robust significantly correlated CG sites (r > 0.8; r < − 0.8; p < 6.8×10^−8^). (C) Heat map of hierarchical clustering 17 paired precancerous/HCC samples using DNA methylation beta values of the 43 robust significantly correlated CG sites. (D) Representative repetition of 10-fold cross-validation LASSO coefficients of 43 candidate CpG sites. (E) LASSO coefficient profiles of the coefficient paths of the 43 CpG sites associated with HCC occurrence.


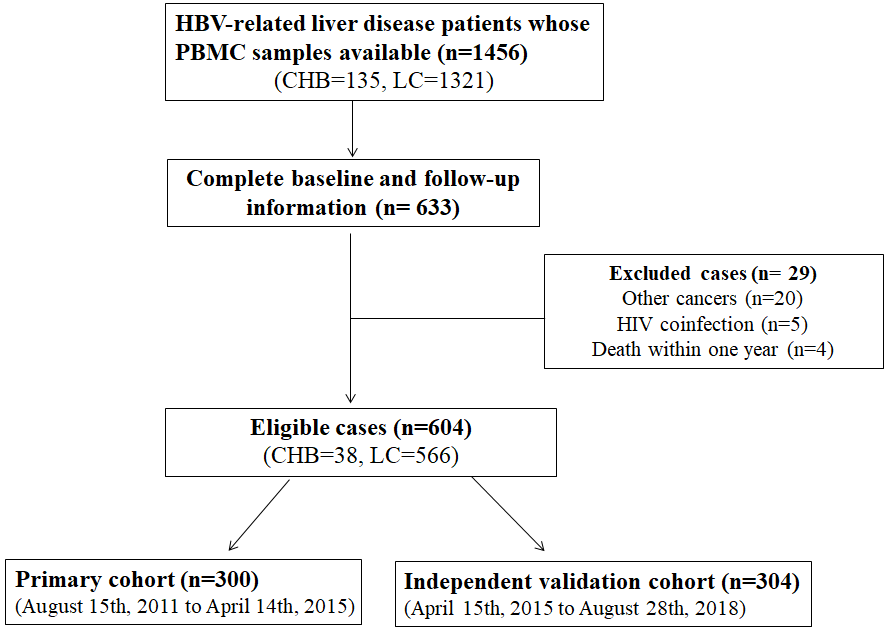
**Figure S6. Flow chart for patient selection.** CHB: chronic hepatitis B; LC: liver cirrhosis; HIV: human immunodeficiency virus.


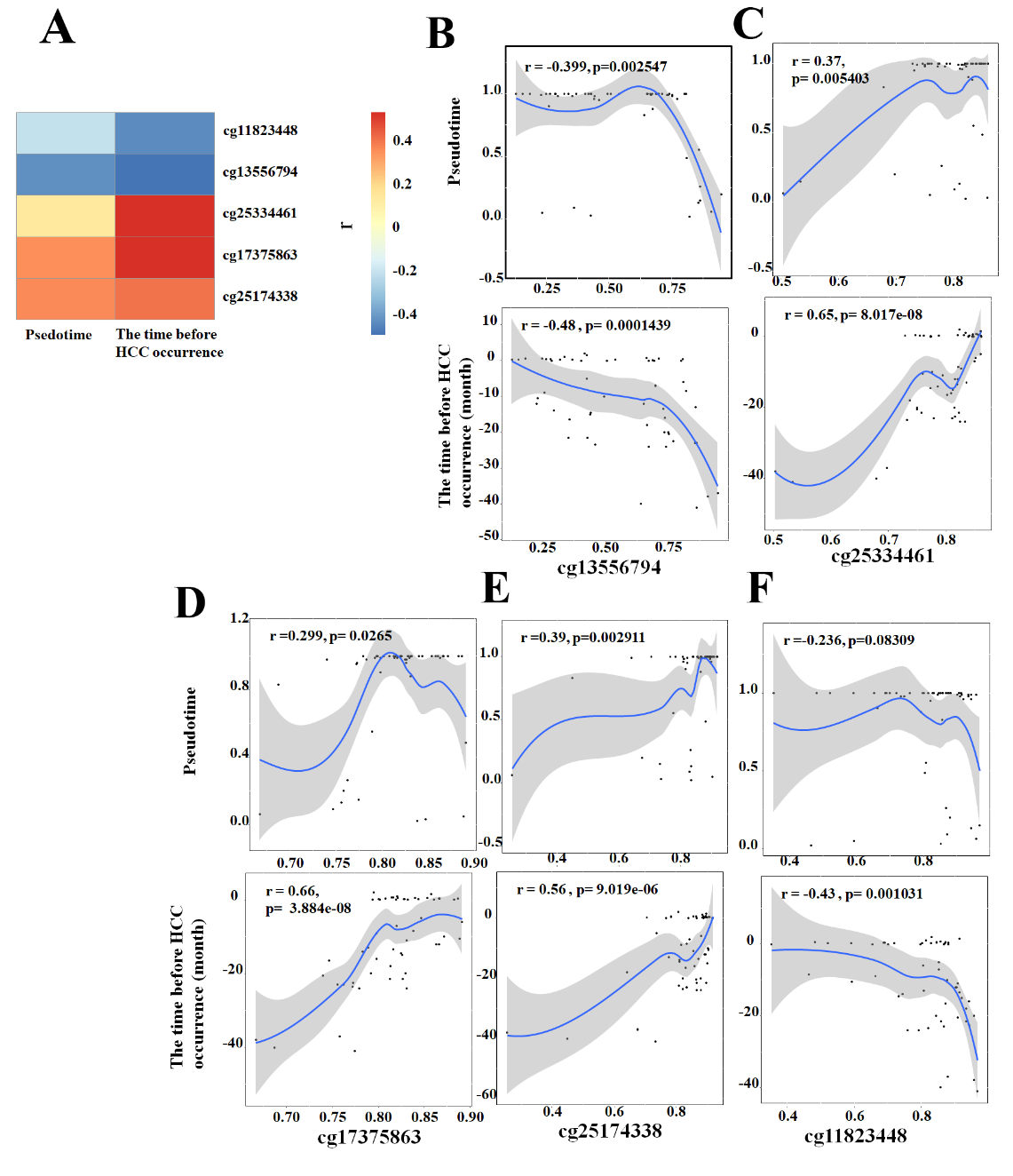


**Figure S7. The five robust significantly correlated CpG sites were evaluate using TimeAx model.** Associations between pseudotime and chronological time with the five CpG sites (cg13556794, cg17375863, cg25174338, cg11823448 and cg25334461) shown as a heatmap (A) and scatterplots (B-F: trend line appears in blue).


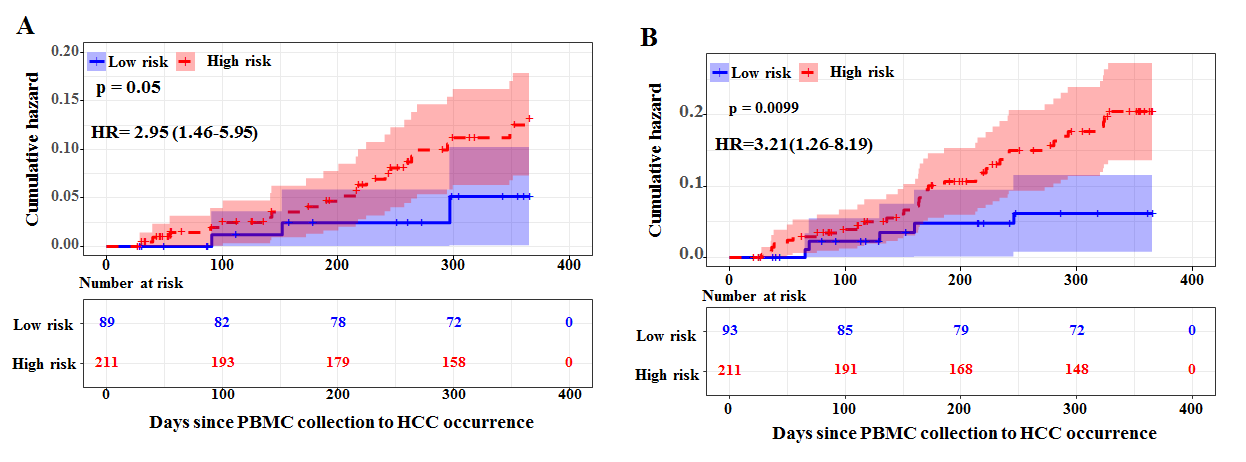


**Figure S8. Kaplan-Meier survival analysis one-year HCC cumulative hazard of low- and high-risk groups defined by five-CpG-classifier in primary cohort (A) and independent validation cohort (B)**. P values were calculated using a log-rank test and hazard ratios using a univariate Cox regression analysis. HR: hazard ratio.


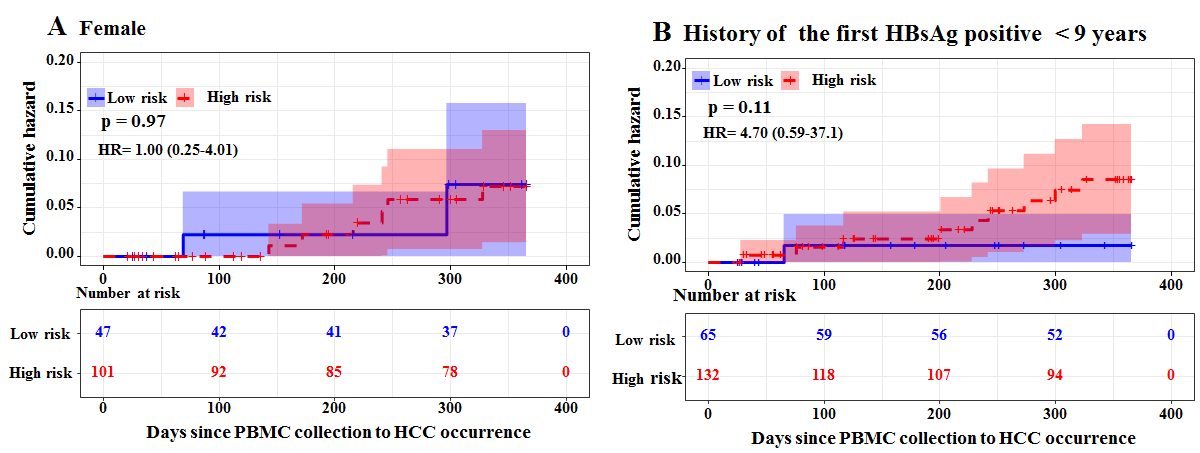
**Figure S9. Five-CpG-classifier was stratified by female (A) and history of the first HBsAg positive < 9 years (B) in whole retrospective cohort.** P values were calculated using a log-rank test and hazard ratios using a univariate Cox regression analysis. HR: hazard ratio.

**Reference**

1.Zhang Y, Petropoulos S, Liu J, et al. The signature of liver cancer in immune cells DNA methylation. Clinical epigenetics 2018; 10(1): 8.
