# Supplementary material for: The methylation signature of hepatocellular carcinoma trajectory based on pseudotime and chronological time for predicting precancerous patients
Source: Oncologist. 2024 Nov 26;30(8):oyae292. doi: 10.1093/oncolo/oyae292 (PMC12395135; doi:10.1093/oncolo/oyae292)
Supplement: oyae292_suppl_Supplementary_Tables [file oyae292_suppl_supplementary_tables.docx]

| Table S1. Stratification analysis of the five-CpG-classifier by clinicopathological risk factors | | |
| --- | --- | --- |
| Clinicopathological risk factors of HBVLD patients (M) | HCC occurred within one year (N) | High-risk patients defined by five-CpG-classifier (n, n/N) |
| Age (≥53 years, M=278) | 40 | 34 (85.00%) |
| Age (<53 years, M=326) | 27 | 24 (88.89%) |
| Sex (Female, M=148) | 9 | 6 (66.7%) |
| Sex (Male, M=456) | 58 | 52 (89.66%) |
| History of the first HBsAg+ (≥ 9 years, M=407) | 57 | 49 (85.96%) |
| History of the first HBsAg+ (< 9 years, M=197) | 10 | 9 (90.00% ) |
| alpha-fetoprotein, (≥7ng/ml, M=180) | 28 | 25 (89.29%) |
| alpha-fetoprotein, (<7ng/ml, M=424) | 39 | 33 (84.62%) |
| PLR (≥53.5, M=464) | 45 | 37(82.22%) |
| PLR (<53.5, M=140) | 22 | 21 (95.45%) |

Note: PLR, the platelet to lymphocyte ratio

| Table S2. Coefficients Cox regression of HCC model for constructing model. | | | | |
| --- | --- | --- | --- | --- |
|  | β | HR (95% CI) | S.E. | p Value |
| Age (years), ≥53 vs<53 | 0.7408 | 2.0976 (1.2756-3.4494) | 0.25377 | 0.0035 |
| Sex (Female vs Male) | -0.8452 | 0.42947 (0.20972-0.87946) | 0.3657 | 0.0208 |
| Alpha-fetoprotein (AFP)  (ng/ml), log_10_ | 0.65794 | 1.9308 (1.1726- 3.1792) | 0.25444 | 0.0097 |
| History of the first HBsAg positive | 1.0639 | 2.8977 (1.4735- 5.6984) | 0.34504 | 0.002 |
| Five-CpG-classifier | 0.99052 | 2.6926 (1.3323- 5.4418) | 0.35898 | 0.0058 |
| PLR | -0.45653 | 0.63348 (0.37451- 1.0715) | 0.26818 | 0.0487 |

Note: S.E. = standard error; HR = hazard ratio; CI = confidence interval, PLR= the platelet to lymphocyte ratio;

| Table S2. Coefficients Cox regression of HCC model for constructing model. | | | | |
| --- | --- | --- | --- | --- |
|  | β | HR (95% CI) | S.E. | p Value |
| Age (years), ≥53 vs<53 | 0.7408 | 2.0976 (1.2756-3.4494) | 0.25377 | 0.0035 |
| Sex (Female vs Male) | -0.8452 | 0.42947 (0.20972-0.87946) | 0.3657 | 0.0208 |
| Alpha-fetoprotein (AFP)  (ng/ml), log_10_ | 0.65794 | 1.9308 (1.1726- 3.1792) | 0.25444 | 0.0097 |
| History of the first HBsAg positive | 1.0639 | 2.8977 (1.4735- 5.6984) | 0.34504 | 0.002 |
| Five-CpG-classifier | 0.99052 | 2.6926 (1.3323- 5.4418) | 0.35898 | 0.0058 |
| PLR | -0.45653 | 0.63348 (0.37451- 1.0715) | 0.26818 | 0.0487 |

Note: S.E. = standard error; HR = hazard ratio; CI = confidence interval, PLR= the platelet to lymphocyte ratio;
